# Adaptive smoothing of multi-shell diffusion weighted magnetic resonance data by msPOAS

**DOI:** 10.1016/j.neuroimage.2014.03.053

**Published:** 2014-07-15

**Authors:** S.M.A. Becker, K. Tabelow, S. Mohammadi, N. Weiskopf, J. Polzehl

**Affiliations:** a Weierstrass Institute for Applied Analysis and Stochastics, Berlin, Germany; b Wellcome Trust Centre for Neuroimaging, UCL Institute of Neurology, London, United Kingdom

## Abstract

We present a novel multi-shell position-orientation adaptive smoothing (msPOAS) method for diffusion weighted magnetic resonance data. Smoothing in voxel and diffusion gradient space is embedded in an iterative adaptive multiscale approach. The adaptive character avoids blurring of the inherent structures and preserves discontinuities. The simultaneous treatment of all *q*-shells improves the stability compared to single-shell approaches such as the original POAS method. The msPOAS implementation simplifies and speeds up calculations, compared to POAS, facilitating its practical application. Simulations and heuristics support the face validity of the technique and its rigorousness. The characteristics of msPOAS were evaluated on single and multi-shell diffusion data of the human brain. Significant reduction in noise while preserving the fine structure was demonstrated for diffusion weighted images, standard DTI analysis and advanced diffusion models such as NODDI. MsPOAS effectively improves the poor signal-to-noise ratio in highly diffusion weighted multi-shell diffusion data, which is required by recent advanced diffusion micro-structure models. We demonstrate the superiority of the new method compared to other advanced denoising methods.

## Highlights

• Method for structure preserving smoothing multi-shell dMRI data
• Does not rely on any dMRI diffusion model
• Outperforms naive single-shell POAS and other approaches
• Feasible for real data application
• Implemented within a freely available package **dti** for the R Language

## Graphical abstract

**Figure:**
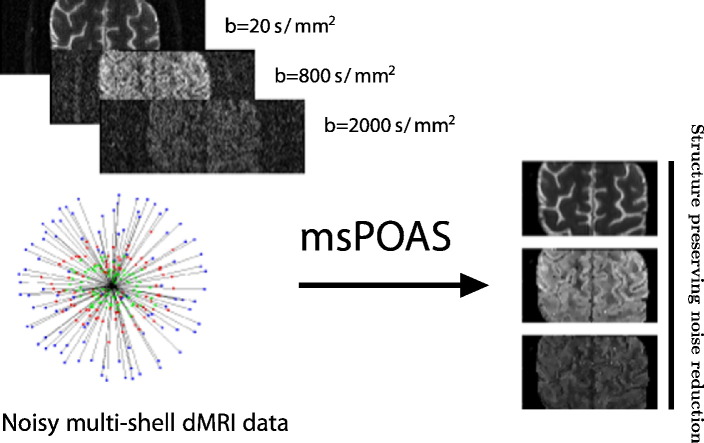

## Introduction

Diffusion weighted magnetic resonance imaging (dMRI) is a versatile tool for in-vivo imaging of anisotropic tissue structure, especially, but not exclusively in the human brain (Jones, 2010, Mori, 2007). The diffusion weighted contrast enables various types of analyses to characterize the brain structure of the normal brain, for a broad range of conditions affecting the brain, or for developmental studies (Johansen-Berg and Behrens, 2009).

DMRI data consist of a series of 3D image volumes acquired by applying diffusion weighting magnetic field gradients in various directions. Depending on the experiment varying gradient strengths or diffusion times, which determine the *b*-value of the measurement, are used (Callaghan, 1991). The diffusion profile obtained from the diffusion images reveals information about the intra-voxel structure, see, e.g., Mitra and Sen (1992). This explains the broad interest in dMRI as it enables measuring tissue properties that exist at the micron level, whereas the voxel size of a dMRI measurement is only at a millimeter level. A wide range of models for the diffusion profile has been developed, such as the diffusion tensor model (DTI) in Basser et al., 1994a, Basser et al., 1994b, tensor mixture models (Assaf and Basser, 2005, Behrens et al., 2003, Tabelow et al., 2012), the orientation distribution function (Tuch, 2004) and higher order tensor models (Liu et al., 2003, Özarslan and Mareci, 2003), to name only a small selection. Some models like DTI can be evaluated based on measurements on a single *q*-shell, i.e., for a single *b*-value, others like Diffusion Kurtosis Imaging (DKI, Jensen et al., 2005, Tabesh et al., 2011), or methods to estimate the full diffusion propagator or its radial part (Aganj et al., 2010, Cheng et al., 2010, Descoteaux et al., 2011, Hosseinbor et al., 2013, Özarslan et al., 2006) require or benefit from multi-shell data.

Although it is generally accepted that models beyond the diffusion tensor are needed to adequately describe complex fiber geometries and compartmentalization in white matter, see e.g. Jones et al. (2013), most dMRI studies still use the simple diffusion tensor model. One reason for this inconsistency in the literature is the simple fact that multi-shell and high angular resolution dMRI data are often mandatory to obtain stable beyond tensor estimates but are expensive in terms of scan time and signal-to-noise ratio (SNR). Moreover, SNR inherently decreases with increasing *b*-value for the diffusion weighted images. Thus, noise hampers modeling for dMRI data in general, but for multi-shell data with high *b*-values in particular.

Another realization in dMRI from the last years was that high spatial resolution improves resolving complex fiber structures (Heidemann et al., 2010, Kamali et al., 2013, Kleinnijenhuis et al., 2012, Zhan et al., 2012). As the increase in spatial resolution also reduces the SNR, this further deteriorates the image quality. In order to reduce noise in dMRI data a number of different approaches have been developed ranging from Gaussian filtering (Westin et al., 1999) over smoothing procedures in tensor space for DTI (Arsigny et al., 2006, Fletcher, 2004) and ODF space for HARDI (Goh et al., 2011) to denoising algorithms based on partial differential equations (Ding et al., 2005, Duits and Franken, 2011, Parker et al., 2000), non-local means (Coupe et al., 2013, Wiest-Daesslé et al., 2008), low-rank approximations of the data (Cauley et al., 2013, Lam et al., 2013), sparsity (Patel et al., 2011), and reconstructions in *k*-space (Haldar et al., 2013) and many others, see also the references in the latter paper.

Recently, we developed a position-orientation adaptive smoothing (POAS) algorithm (Becker et al., 2012) based on the propagation-separation approach (Becker and Mathé, 2013, Polzehl and Spokoiny, 2006). The method directly smooths diffusion weighted images measured on a single *q*-shell. It is applied to the dMRI data prior to any modeling. Hence, it does not introduce a model-specific bias into the data and any model for dMRI data may be used after smoothing. The gain in SNR is quite substantial due to the fact that the algorithm considers the geometric properties of the measurement space. Each signal value in a diffusion weighted image is associated to its position in (voxel) space and the (diffusion sensitizing) orientation. Here, ℝ^3^ stands for the 3D voxel position space and $S^{2}$ for the unit sphere, where the diffusion gradient orientations are distributed. The measurement space can thus be described by a combined space ${\mathbb{R}}^{3}\times S^{2}$. In Becker et al. (2012), POAS has been proven to be able to reduce noise in dMRI data without blurring the structural borders in the images which is a result of its adaptive properties.

This article aims to substantiate and extend the original POAS proposal in several very important directions. We will start with the extension to *multi-shell data* as required by higher order diffusion models. Basically, the POAS method could be applied separately for each *b*-value. However, at high *b*-values and high spatial resolution the loss in SNR inhibits POAS to adapt to smaller structures (as will be shown). Here, we present an extension of the POAS method by allowing for simultaneous smoothing on all *q*-shells. We denote this generalization multi-shell POAS (msPOAS). The new algorithm uses the geometry of the measurement space and the relatedness of observed values on different shells.

In addition to the multi-shell extension, significant simplifications to the algorithm in comparison to the single-shell method accelerate it and improve its practical feasibility. We will present some heuristics and simulations which support the generalizability of the theoretical properties in Becker and Mathé (2013) to msPOAS. Finally, we evaluate msPOAS in very high resolution multi-shell and single-shell dMRI data at 3 T and 7 T and provide recommendations how to use the method.

## Theory

When reconstructing a dMRI-scan, we do not directly observe the image, which we are interested in. The scanner yields complex-valued data in *k*-space, which relate to the signal attenuation due to water diffusion. For typical Cartesian *k*-space acquisitions the data are transformed via inverse Fourier transformation to a diffusion weighted image (Callaghan, 1991). In case of multi-channel RF coils (Roemer et al., 1990), this image is reconstructed from the data of all coils. The details depend on the acquisition and image reconstruction methods among which the most popular ones are SENSE (Pruessmann et al., 1999) and GRAPPA (Griswold et al., 2002) for parallel imaging or their non accelerated analogues. The complex-valued signal in image space is typically transformed to real positive numbers by extracting the magnitude image and neglecting the phase component. The data are generally pre-processed to compensate for artifacts due to motion (Mohammadi et al., 2013, Storey et al., 2007), magnetic field inhomogeneities (Andersson et al., 2003, Mohammadi et al., 2012, Ruthotto et al., 2012), eddy currents (Andersson and Skare, 2002, Jezzard et al., 1998, Mohammadi et al., 2010) or noise of different origins. The corresponding methods are applied at different points of the processing pipeline. Our msPOAS method will be directly applied to the reconstructed diffusion weighted images to improve and stabilize the subsequent modeling and analysis of the data. Ideally, it should be executed after all other pre-processing steps. However, other smoothing should not be performed prior to msPOAS.

### Concept of multi-shell position-orientation adaptive smoothing (msPOAS)

#### Position-orientation space

MsPOAS is a smoothing procedure in position-orientation space, i.e. the entire (5-dimensional) measurement space ${\mathbb{R}}^{3}\times S^{2}$ of dMRI multi-shell data, which is formed by the (voxel) position $\vec{v}\in {\mathbb{R}}^{3}$ and the (diffusion sensitizing) orientation $\vec{g}\in S^{2}$. Compared to standard 3D-adaptive smoothing approaches it gains its strength from the richer vicinity structure in this higher dimensional space. The extra two dimensions substantially increase the amount of data that can potentially be pooled within the weighted averages used in msPOAS and thus increase power of the method.

#### Adaptation

MsPOAS uses weighted means of image intensity values of neighboring design points, characterized by voxel position and gradient orientation, to infer on the expected signal. The adaptation of the weighting schemes to the inherent intensity structure is established in an iterative manner. At each iteration step the estimated signal intensity at neighboring design points is compared to the intensity at the current position: An observation at a neighboring point is excluded from the weighted average, i.e., a zero weight is assigned, if the estimated signal intensities are significantly different. Using this adaptive weighting scheme a new estimated signal intensity is calculated for the design point under consideration. This is conducted for every design point. With each new iteration, a bandwidth in the design space is increased, i.e., the vicinity of candidate points is enlarged, permitting the higher variance reduction and increased stability of estimated intensities. Decisions on signal intensities being significantly different get more informative and smaller deviations from a homogeneous intensity structure can be detected. This establishes a natural, data-driven choice of adaptive weighting schemes and ensures an optimum relation between noise reduction and adaptation to the underlying structure.

#### Extension to multiple shells

The performance of msPOAS is further improved by simultaneous utilization of all diffusion shells to determine the adaptive weighting schemes. Information from different shells is combined under the assumption that intensity deviations on any shell indicate an inhomogeneity of the inherent structure. In this way, the determination of the weighting schemes can benefit from the high-SNR, low-diffusion-weighting images with low orientation contrast in position space, and from the low-SNR, high-diffusion-weighting images with high orientation contrast in orientation space.

### Detailed outline of msPOAS

#### Description of the data

In multi-shell dMRI, one measures values on a regular grid of voxel $\vec{v}\in V\subseteq {\mathbb{R}}^{3}$ for a number B of *b*-values, *b* > 0, from a set *B*. For each *b*-value, i.e., for each shell, data $S_{b}\left(\vec{v},\vec{g}\right)$ is acquired with varying diffusion gradients $\vec{g}$ from a set *G*_*b*_, which might depend on the shell. These can be identified with elements of the 2-sphere such that $G_{b}\subseteq S^{2}:=\left\{\vec{g}\in {\mathbb{R}}^{3}:\left\|\vec{g}\right\|=1\right\}$.

Additionally, at least one non-diffusion weighted image *S*_0_ is acquired. In case of several *S*_0_-images, we consider the corresponding mean image ${\hat{S}}_{0}$. However, for better readability we will use *S*_0_ instead of ${\hat{S}}_{0}$ for notation. In the following, we interpret the non-weighted data *S*_0_ as a shell with *b* = 0 and denote the set of *b*-values including *b* = 0 by *B*_0_ := *B* ∪ {0} and the (artificial) gradient set by $G_{0}:=\left\{\vec{0}\right\}$. Furthermore, we set $S_{0}\left(\vec{v},\vec{0}\right):=S_{0}\left(\vec{v}\right)$ for a common notation of all signal values.

**Fig. 1:**
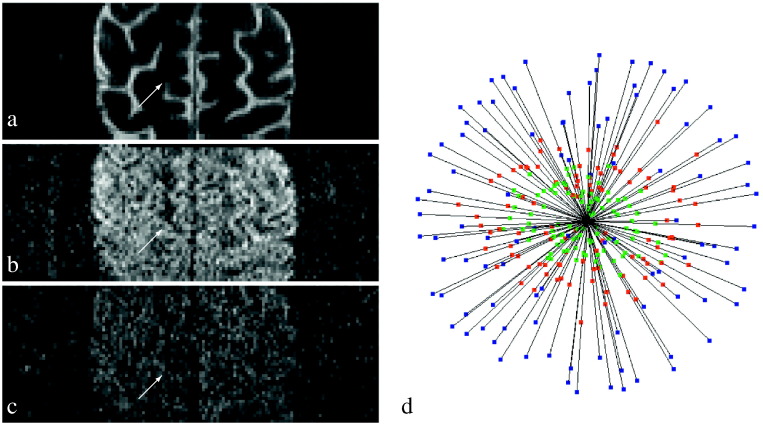
Diffusion weighted data *S* for an arbitrarily selected slice of the multi-shell data described in Methods. a) Slice of the non-diffusion weighted data. b) Same slice taken with diffusion-weighting gradient $\vec{g}\in S^{2}$ and *b*-value *b* = 800s/mm^2^. c) Same slice taken with the same gradient $\vec{g}$ and *b*-value *b* = 2000s/mm^2^. The intensity of the diffusion weighted images in (b + c) has been up-weighted to make all three images visually feasible at once. d) Shows the data within a single voxel (see arrow) as a 3D plot for all measured diffusion gradients in red and green at *b* = 800s/mm^2^ and *b* = 2000s/mm^2^, respectively. For comparison the non-diffusion weighted value is repeatedly shown as blue point for each gradient. The distance of the points to the center of the sphere is the corresponding signal value. Each 3D diffusion weighted image (a–c) is fully described by the set ${\left\{S_{b}\left(\vec{v},\vec{g}\right)\right\}}_{\vec{v}\in V}\subseteq \mathbb{R}$ of signals in voxel space for a fixed diffusion gradient direction $\vec{g}\in G$ (if *b* ≠ 0) and the *b*-value *b* ∈ *B*_0_. Conversely, the data in a single voxel $\vec{v}\in V$ equals the set ${\left\{S\left(\vec{v},\vec{g}\right)\right\}}_{\vec{g}\in G}\subseteq {\mathbb{R}}^{B+1}$ of vectors, see (d).

For msPOAS we view the data as possibly incomplete observations of a vector-valued function S defined on the measurement space $V\times G\subseteq {\mathbb{R}}^{3}\times S^{2}$ by

(1) $$S:V\times G∍\left(\vec{v},\vec{g}\right)\mapsto {\left(S_{0}\left(\vec{v}\right),S_{b_{1}}\left(\vec{v},\vec{g}\right),\ldots ,S_{b_{B}}\left(\vec{v},\vec{g}\right)\right)}^{T}\in {\mathbb{R}}^{B+1},$$

where *G* is the set of all gradient directions measured on any shell including the artificial direction $\vec{0}$. The vector data structure is visualized in Fig. 1. Before this function S is well-defined two additional cases have to be considered.

First, if the gradient schemes *G*_*b*_ do not coincide for all measured *b*-values the vector in Eq. (1) will not be completely observed. We then fill the missing values $S_{b}\left(\vec{v},\vec{g}\right)$, for every *b* ∈ *B* with $\vec{g}\notin G_{b}$ by interpolated values $S_{b}\left(\vec{v},\vec{g}\right)$ using spherical interpolation.

Second, for $\vec{g}=\vec{0}$ the vector in Eq. (1) is incomplete as well. We therefore define mean values of the signal on the corresponding shell as

$$S_{b}\left(\vec{v},\vec{0}\right):={\left|G_{b}\right|}^{-1}\sum_{\vec{g}\in G_{b}}S_{b}\left(\vec{v},\vec{g}\right),$$

where |*G*_*b*_| is the number of gradients measured on the shell with *b*-value *b*. This enables estimation of expected *S*_0_ -images in msPOAS. For details on the spherical interpolation and its application to the signal vector in Eq. (1), see Appendix A.

For simplicity, we do in the following no longer distinguish between the original data *S* and the interpolated data S denoting both by *S*. Additionally, we apply the interpolation (Appendix A.1) to the adaptive estimates ${\tilde{S}}_{b}^{\left(k\right)}$ defined below, replacing *S*_*b*_ by ${\tilde{S}}_{b}^{\left(k\right)}$ in the respective formulas.

#### Initial parameter choices of the algorithm

MsPOAS uses several pre-specified quantities. The adaptive weights are defined as the product of two kernel functions *K*_loc_ and *K*_ad_. The specific choice *K*_loc_ is not crucial, see e.g. Scott (1992, Section 6.2.3). Both kernels should be non-increasing with support [0,1). In our implementation of the algorithm we use, because of their statistical and numeric efficiency,

(2) $$K_{\mathrm{loc}}\left(x\right):={\left(1-x^{2}\right)}_{+}\;\mathrm{and}\;K_{\mathrm{ad}}\left(x\right):=\left\{\begin{array}{cc} \begin{array}{l} 1\\ {\left(2-2x\right)}_{+} \end{array} & \begin{array}{l} \mathrm{for}\;0\leq x<0.5,\\ \mathrm{for}\;0.5\geq x. \end{array} \end{array}\right\}$$

In the arguments of both kernels as considered later, a bandwidth controls the amount of information that is taken into account. The bandwidth λ > 0 that will be used in the argument of *K*_ad_ determines the amount of adaptivity. As described in Becker et al. (2013, Section 2.5), it can be chosen independently of the data at hand by simulation. We give some more details about this choice in the section Choice of parameters for msPOAS. Depending on the gradient scheme, we use a pre-specified sequence of increasing bandwidths ${\left\{h^{\left(k\right)}\right\}}_{k=0}^{k^{*}}$ with *h*^(0)^ > 0 in the argument of *K*_loc_. The precise choice which is used in our implementation is given in Appendix B.

Furthermore, we fix some appropriate metric $\delta_{\kappa }:\left({\mathbb{R}}^{3}\times S^{2}\right)\times \left({\mathbb{R}}^{3}\times S^{2}\right)\to \left[0,\infty \right)$ on the measurement space ${\mathbb{R}}^{3}\times S^{2}$, which is used to combine the spatial and spherical diffusion information. Instead of the discrepancy proposed in Becker et al. (2012) we decided to use the metric

(3) $$\delta_{\kappa }\left(m_{1},m_{2}\right):=\left\|{\vec{v}}_{1}-{\vec{v}}_{2}\right\|+\kappa^{-1}\arccos\left|\langle {\vec{g}}_{1},{\vec{g}}_{2}\rangle \right|,$$

for all $m_{l}=\left({\vec{v}}_{l},{\vec{g}}_{l}\right)\in {\mathbb{R}}^{3}\times S^{2}$, *l* = 1,2, see Hagmann et al. (2006). This simplified metric provides left-invariance of msPOAS, as proven in Becker et al. (2013, Section 2.6). In order to identify each gradient $\vec{g}\in S^{2}$ with the opposite direction $-\vec{g}$ we consider the absolute value of the scalar product $\langle {\vec{g}}_{1},{\vec{g}}_{2}\rangle$. This is in accordance with the symmetry of the diffusion process.

The parameter *κ* of the discrepancy *δ*_*κ*_(.,.) balances between spatial and spherical smoothing in the voxel space *V* ⊆ ℝ^3^ and the gradient space $G\subseteq S^{2}$. We set *κ*^(*k*)^ := *κ*_0_/*h*^(*k*)^ at iteration step *k* as already proposed in Becker et al. (2012, Section 2.5). The precise choice of the initial value *κ*_0_ depends on the data at hand as we discuss in the section Choice of parameters for msPOAS.

#### The non-adaptive weights and the location bandwidths

For each *b* ∈ *B*_0_ and all design points *m* ∈ *V* × *G*_*b*_ we introduce the non-adaptive estimator

$${\bar{S}}_{b}^{\left(k\right)}\left(m\right)=\sum_{n\in V\times G_{b}}{\bar{w}}_{\mathrm{mn}}^{\left(k\right)}S_{b}\left(n\right)/{\bar{N}}_{m,b}^{\left(k\right)}\in \mathbb{R}$$

with

$${\bar{w}}_{\mathrm{mn}}^{\left(k\right)}:=K_{\mathrm{loc}}\left(\delta_{\kappa }\left(m,n\right)/h^{\left(k\right)}\right)\;\mathrm{and}\;{\bar{N}}_{m,b}^{\left(k\right)}:=\sum_{n\in V\times G_{b}}{\bar{w}}_{\mathrm{mn}}^{\left(k\right)}.$$

This will be used for initialization. For *b* = 0 the locations *m*,*n* ∈ *V* × *G*_0_ do not carry directional information. In this case, *δ*_*κ*_(*m*,*n*) can be replaced by the Euclidean distance $\left\|{\vec{v}}_{m}-{\vec{v}}_{n}\right\|$ in voxel space.

#### The adaptive weights

For the adaptive estimator, we take advantage of the whole information in the data vector given in Eq. (1). This requires an appropriate modification of the statistical penalty used for POAS, see Becker et al. (2012), and the specification of the probability distribution of the observations, up to some parameter *θ*. We assume the standardized signal $S_{b}\left(\vec{v},\vec{g}\right)/\sigma$ to be non-central *χ* distributed

(4) $$S_{b}\left(\vec{v},\vec{g}\right)/\sigma ∼\chi_{2L^{\prime }}\left(\theta \right),$$

with 2*L*′ degrees of freedom and non-centrality parameter *θ*. Here, *L*′ ∈ ℕ denotes the effective number of RF receiver coils and *σ* > 0 is the noise standardized deviation. For a detailed discussion of this assumption, see for example Thunberg et al. (2007), Dietrich et al. (2008), Aja-Fernández et al. (2011), Sotiropoulos et al. (2013), Hutton et al. (2012), Becker et al. (2013). *L*′ and *σ* depend on the image reconstruction method. Although msPOAS uses a homogeneous *σ* it could be easily adapted for spatially varying noise standard deviation.

As in (Becker et al., 2013), we define the statistical penalty for msPOAS as

(5) $$s_{\mathrm{mn}}^{\left(k\right)}:=\sum_{b\in B_{0}}\;{\tilde{N}}_{m,b}^{\left(k-1\right)}KL\left(\frac{{\tilde{S}}_{b}^{\left(k-1\right)}\left(m\right)}{\hat{\sigma }},\frac{{\tilde{S}}_{b}^{\left(k-1\right)}\left(n\right)}{\hat{\sigma }}\right),$$

where KL denotes the Kullback–Leibler divergence of two non-central *χ*-distributions with respective expectation values. See Appendix C for its calculation. Here, ${\tilde{N}}_{m,b}^{\left(k-1\right)}$ (see Appendix B) relates for each *b*-value to the achieved variance reduction using the adaptive weights

(6) $${\tilde{w}}_{\mathrm{mn}}^{\left(k\right)}:={\bar{w}}_{\mathrm{mn}}^{\left(k\right)}\cdot K_{\mathrm{ad}}\left(s_{\mathrm{mn}}^{\left(k\right)}/\lambda \right),$$

where *K*_ad_ and λ are defined as in the section Initial parameter choices of the algorithm. The adaptive estimator is then

$${\tilde{S}}_{b}^{\left(k\right)}\left(m\right)=\sum_{n\in V\times G_{b}}\;{\tilde{w}}_{\mathrm{mn}}^{\left(k\right)}S_{b}\left(n\right)/{\tilde{N}}_{m,b}^{\left(k\right)}.$$

#### The algorithm

Finally, we summarize the algorithm for multi-shell position-orientation adaptive smoothing (msPOAS). The initialization of the algorithm is done using the non-adaptive estimator for *k* = 0 (*h*^(0)^ = 1). Iteration is performed for *k* steps until some final number of steps *k*^⋆^ is reached.

• Input parameters: Sequence of location bandwidths ${\left\{h^{\left(k\right)}\right\}}_{k=0}^{k^{*}}$, balancing parameter *κ*^(*k*)^, adaptation parameter λ > 0.
• Initialization: ${\tilde{S}}_{b}^{\left(0\right)}\left(m\right):={\bar{S}}_{b}^{\left(0\right)}\left(m\right)$ and ${\tilde{N}}_{m,b}^{\left(0\right)}:={\bar{N}}_{m,b}^{\left(0\right)}$ for all *m* ∈ *V* × *G*_*b*_, *b* ∈ *B*_0_.
• Iteration: For each *b* ∈ *B*_0_ and $m:=\left({\vec{v}}_{m},{\vec{g}}_{m}\right)\in V\times G_{b}$ do the following. Interpolate the missing values of ${\tilde{S}}_{b^{\prime }}^{\left(k-1\right)}\left(m\right)$ and ${\tilde{N}}_{m,b^{\prime }}^{\left(k\right)}$, *b*′ ∈ *B* ∖ {*b*}, according to Eqs. (A.1), (A.2). Then, calculate the statistical penalty
  $$s_{\mathrm{mn}}^{\left(k\right)}=\sum_{b\in B_{0}}\;{\tilde{N}}_{m,b}^{\left(k-1\right)}KL\left(\frac{{\tilde{S}}_{b}^{\left(k-1\right)}\left(m\right)}{\hat{\sigma }},\frac{{\tilde{S}}_{b}^{\left(k-1\right)}\left(n\right)}{\hat{\sigma }}\right)$$
  and the adaptive weights
  $${\tilde{w}}_{\mathrm{mn}}^{\left(k\right)}=K_{\mathrm{loc}}\left(\delta_{\kappa^{\left(k\right)}}\left(m,n\right)/h^{\left(k\right)}\right)\cdot K_{\mathrm{ad}}\left(s_{\mathrm{mn}}^{\left(k\right)}/\lambda \right)$$
  for *n* ∈ *V* × *G*_*b*_, the corresponding sum over the adaptive weights
  $${\tilde{N}}_{m,b}^{\left(k\right)}=\max_{k^{\prime }\leq k}\left(\sum_{n\in V\times G_{b}}{\tilde{w}}_{\mathrm{mn}}^{\left(k^{\prime }\right)}\right),$$
  and the adaptive estimator
  $${\tilde{S}}_{b}^{\left(k\right)}\left(m\right)=\sum_{n\in V\times G_{b}}\;{\tilde{w}}_{\mathrm{mn}}^{\left(k\right)}S_{b}\left(n\right)/\sum_{n\in V\times G_{b}}{\tilde{w}}_{\mathrm{mn}}^{\left(k\right)}.$$
• Stopping: Stop if *k* = *k*^∗^ and return ${\tilde{S}}_{b}^{\left(k^{*}\right)}\left(m\right)$ for each *b* ∈ *B*_0_ and all *m* ∈ *V* × *G*_*b*_, else set *k* := *k* + 1.

**Fig. 2:**
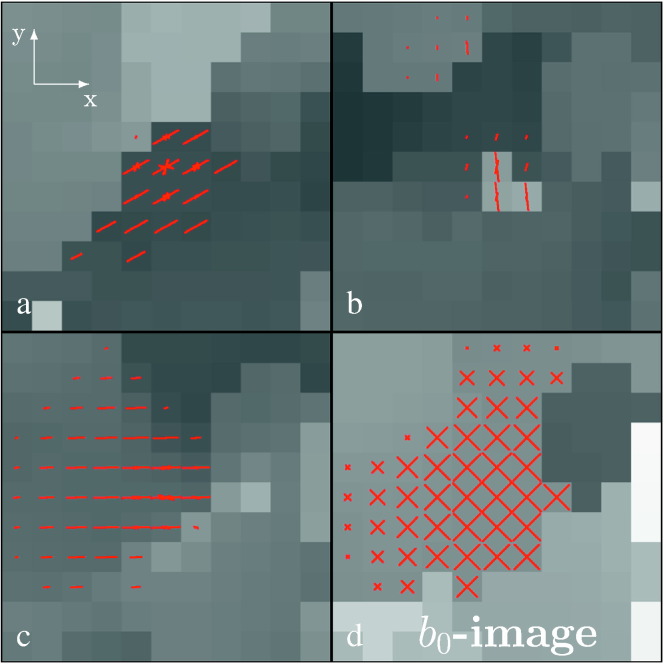
Adaptive weighting schemes obtained for the single-shell dataset used in this paper at iteration step *k* = 20. Weights are shown for the same voxel position of three diffusion weighted images (a to c) with different gradients and one *b*_0_-image (d). The length of the lines (size of the cross for the *b*_0_-image) corresponds to the size of the weights, its direction corresponds to the gradient direction projected onto the *xy*-plane. Non-zero weights also occur for different directions, see for example the central voxel in a). Weights for points in different slices are not shown. Illustrations are overlayed on estimated image intensities from the previous iteration centered at the voxel of interest. For an animated movie showing the development of the weights over iterations for non-adaptive and adaptive smoothing, see the online version of this paper.

In Fig. 2 we illustrate adaptive weighting schemes obtained for the single-shell dataset at iteration step *k* = 20.

## Methods

### Simulations

In order to provide some more intuition for the behavior of the algorithm, we show two simple examples on a one-dimensional design $X:=\left\{1,\ldots ,4000\right\}$. Here, we used the **R**-package **aws** (Polzehl, 2013), where the propagation-separation approach has been implemented for one-, two- and three-dimensional designs for many classes of distributions of the observations including those considered in this paper for msPOAS. We consider a piecewise constant test function *θ*_1_(.) and a piecewise polynomial test function *θ*_2_(.). Then, we simulate observations that follow a non-central chi-distribution with non-centrality parameter *θ*_*l*_(.), *l* = 1,2 and 2*L*′ = 4 degrees of freedom, i.e., *Y*_*i*_ ∼ *χ*_4_(*θ*_*l*_(*X*_*i*_)) for all $X_{i}\in X$. Smoothed results were provided by the function **aws**() setting $\mathrm{hmax}:=h^{\left(k^{*}\right)}:=4000$ and l kern = “Triangle” using the same kernel functions as in Eq. (2) and setting λ = 20. The values of the considered test functions compare well with the parameters in our real data, see the section Experimental data. Similar situations have been already considered in Becker and Mathé (2013) for Gaussian distributed noise. However, until now it was not clear that these results remain valid for *χ*-distributed observations as the theoretical results in Becker and Mathé (2013) were restricted to one-parameter exponential family distributions.

### Experimental data

#### Dataset 1

We first re-analyzed the dataset from a whole body 7 T MAGNETOM scanner (Siemens Healthcare) used and described already in Becker et al. (2012). The scanner was equipped with gradients with a peak amplitude of 70mT/m and a maximum slew rate of 200 T/m/s (SC72, Siemens Healthcare, Erlangen, Germany). Diffusion weighting gradients were applied along 60 different directions at a *b*-value of 1000 s/mm^2^. 7 interspersed non-diffusion weighted *S*_0_ images were acquired. The scan was repeated 4 times. An optimized monopolar Stejskal–Tanner sequence according to Morelli et al. (2010) together with the ZOOPPA approach described in Heidemann et al. (2012) has been used for the scan. The experiment was performed using a single channel transmit, 24-channel receive phased array head coil (Nova Medical, Wilmington, MA, USA). 91 slices with 10% overlap were acquired at a field-of-view (FoV) of 143 × 147 mm^2^ resulting in an isotropic high resolution of 800 μm. Further imaging protocol parameters were: TR 14.1 s, TE 65 ms, BW 1132Hz/pixel, ZOOPPA acceleration factor of 4.6. A healthy adult volunteer was scanned four times using this protocol in one session after obtaining written informed consent in accordance with the ethical approval from the University of Leipzig. Total acquisition time was 65 min. We used the raw diffusion weighted data.

#### Dataset 2

The second example data was acquired on a 3 T MAGNETOM Trio scanner (Siemens Healthcare) using a reduced FoV-technique as described in Heidemann et al. (2010). The FoV was 161 × 58 mm centered about the motor cortex resulting in an isotropic in-plane resolution of 1.2 mm. 34 axial slices with 1.2 mm slice thickness and 10% gap were acquired. Diffusion weighted data were acquired at 2 different *b*-values: *b* = 800 s/mm^2^ and *b* = 2000s/mm^2^ each with 100 different diffusion gradient directions as suggested by Caruyer et al. (2011). 20 interspersed *S*_0_-images at *b* = 0 s/mm^2^ were acquired with TR 6.1 s, TE 97 ms. One healthy adult volunteer participated in the study approved by the local ethics committee after giving written informed consent. The total scan time was 22 min.

#### Dataset 3

Using the same specifications an additional double-shell dataset was acquired, with 10 replicated measurements of five selected diffusion weighted images for a high SNR reference.

#### Dataset 4

In order to illustrate the properties of the method in the case of experimental data with more and higher *b*-values a fourth dataset was acquired on a 3 T MAGNETOM Trio scanner (Siemens Healthcare) using a reduced FoV-technique as described in Heidemann et al. (2010). The FoV was 156 × 60 mm centered about the motor cortex resulting in an isotropic in-plane resolution of 1.42 mm. 34 axial slices with 1.4 mm slice thickness and 10% gap were acquired. Diffusion weighted data were acquired at 3 different *b*-values: *b* = 800 s/mm^2^, *b* = 2000s/mm^2^ and *b* = 3000 s/mm^2^ each with 70 gradient directions as suggested by Caruyer et al. (2011). 21 interspersed *S*_0_-images at *b* = 0 s/mm^2^ were acquired with TR 6.2 s, TE 108 ms. One healthy adult volunteer participated in the study approved by the local ethics committee after giving written informed consent. The total scan time was 24 min.

### Parameter choices for msPOAS

All datasets were smoothed using the msPOAS method described in this article. The adaptation bandwidth λ of the procedure was fixed at a value of 15 (single-shell) and 20 (multi-shell). The number of iteration steps was *k*^⋆^ = 12 for all datasets for a suitable balance between computation time and amount of achieved smoothness.

The number *L*′ of effective coils, that determines the degrees of freedom of the non-central *χ*-distribution, is usually difficult to estimate from the data. Fortunately, msPOAS is relatively robust against misspecification of *L*′. We therefore used *L*′ = 2 for the single-shell dataset (No. 1) to mimic an average influence of two coils to the resulting distribution, and *L*′ = 1,4,16 for the second dataset (No. 2) to analyze the dependence of the results on the choice of *L*′. We did not explicitly analyze the dependence of the results on the choice of the noise variance *σ* or on the adaptation bandwidth λ as the qualitative behavior can be extracted from the definition of the adaptive weights in Eq. (6) — see also the discussion in sections on Estimation of data-dependent parameters and Choice of parameters for msPOAS, Estimation of data-dependent parameters and Choice of parameters for msPOAS. The balancing parameter *κ*_0_ was 0.5 for the first and 0.3 for the second dataset.

**Fig. 3:**
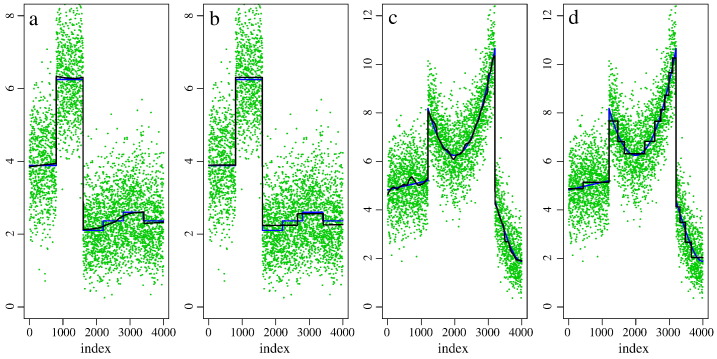
Univariate examples for adaptive smoothing of non-central *χ*-distributed observations for an optimal and for an extremely large bandwidth. From left to right: Simulated data based on local constant non-centrality parameter (NCP), *θ*_1_(.), smoothed with location bandwidths *h*_1_ = 485,3610, and simulated data with locally smooth NCP, *θ*_2_(.), smoothed with location bandwidths *h*_2_ = 81,3610. Observations are presented as green dots, the expected values as blue lines, smoothing results are shown as black lines.

We estimated the noise standard deviation *σ* using the method described in Becker et al. (2012) and the methods “Bk-M1-*χ*” and “Bk-M2-*χ*” described in Aja-Fernández et al. (2009). The estimates for *σ* from the former method are rather independent of *L*′. For the dataset acquired at 7 T (No. 1) we consistently got values around 75, for the first double (No. 2) and the triple-shell datasets acquired at 3 T (No. 4) values around 30 were estimated. The results from the latter two methods depended on *L*′. However, in view of the uncertainty of the estimation of *L*′ and based on our experience with variance estimation in general we decided to use *σ* = 75 for the single-shell data and *σ* = 30 for the double and triple-shell data (No. 2 and 4) for our calculations. The estimated noise level for the double-shell dataset (3 T) with replicated volumes (No. 3) was *σ* = 40. Finally we note that based on these estimates for *σ* the non-centrality parameter of the considered *χ*-distributions is in the range between 0 and 8, therefore we considered these values in the simulated univariate example, see Fig. 3.

### Comparison with single-shell method

For the first (single-shell) dataset (No. 1) we explicitly compared the msPOAS results with the results of POAS obtained in Becker et al. (2012). There, we used λ = 10, *κ*_0_ = 0.6, *σ* = 66, and *L*′ = 2 in the corresponding algorithm. The different choices of the adaptation bandwidth λ in POAS and msPOAS may result from the explicit coupling of the *S*_0_-images in the adaptation. The simplified discrepancy and the new approximation of the Kullback–Leibler divergence should have only a minor impact.

For the second dataset (No. 2) we compared msPOAS with the results of a simple approach that applies POAS separately to each shell of the double-shell dataset.

For the POAS method we used the same variance estimate *σ* = 30 as for msPOAS and a medium *L*′ = 4. For a fair comparison, we chose an adaptation parameter λ for POAS providing similar results as the msPOAS approach for the shell at *b* = 800 s/mm^2^, i.e., λ = 6. The balancing parameter *κ*_0_ was chosen as 0.3. The recombined smoothed diffusion weighted data again forms a double-shell dataset.

### Comparison with other smoothing methods

We compared the msPOAS results with the results of two other smoothing methods suitable for dMRI data in general and for data measured at multiple *b*-values in particular using dataset No. 3. More specifically, we used a non-local means (NLM) method described in Wiest-Daesslé et al. (2008) and a joint denoising method of all diffusion images as reported in Haldar et al. (2013), Varadarajan and Haldar (2013). For both methods implementations are freely available for MATLAB.

For NLM the suggested standard parameters of the method worked well and were chosen for analysis. For the method from Haldar et al. (2013), Varadarajan and Haldar (2013) we had to adjust the standard suggestions to achieve optimal results. Finally we chose *ξ* = 1, λ = 5. Note, that the meaning of this parameter λ is completely different from the corresponding value in msPOAS.

### Analysis of the smoothed datasets

For the first two datasets (Nos. 1 and 2) we estimated the diffusion tensor model, as it is the most widely used in practice and was also used in our previous report (Becker et al., 2012). The diffusion tensors were estimated using the non-linear method described in Polzehl and Tabelow (2009). Then, for the single-shell dataset (No. 1) FA maps were calculated based on the tensor estimates. For the multi-shell dataset (No. 2) we also calculated fiber tracks using a streamline FACT algorithm (Mori et al., 1999) based on diffusion tensor modeling.

For the single-shell dataset (No. 1) we calculated the orientation distribution function (ODF) following Descoteaux et al. (2007) using no regularization and spherical harmonics up to sixth order.

Additionally, for the first double-shell dataset (No. 2) we estimated a one-stick-one-ball model (Behrens et al., 2003) to evaluate the variability of directional estimates in a model that goes beyond the diffusion tensor model. To do this we used FSL (Smith et al., 2004) and sampled 50 directions for the sticks. The model was evaluated for the original data, for the msPOAS result, and for the result from the POAS approach.

We analyzed the first double-shell (No. 2) and the triple-shell dataset (No. 4) using the NODDI model (Zhang et al., 2012). In order to create a more complicated situation from the triple-shell dataset (with lower SNR and more uneven sampling), we also considered a sub-dataset of the triple-shell data by dropping the data from the *b* = 2000s/mm^2^-shell.

### Software

For pre-processing of the multi-shell data, i.e., motion and eddy-current correction, we used SPM (Friston et al., 2006) and the ACID-toolbox (Mohammadi et al., 2010) (http://www.diffusiontools.com/). In particular, we registered the ten replicated measurements in the second double-shell dataset (No. 3) to its corresponding volume in the dataset using this toolbox.

The computations for msPOAS and POAS, as well as the diffusion tensor estimates, FA maps, and fiber tracks, were performed with our **R**-package **dti** (Tabelow and Polzehl, 2013) (version 1.1-5). This package is freely available on CRAN (http://cran.r-project.org). A detailed description of the usage of the package **dti** can be found in Polzehl and Tabelow (2011). The implementation uses FORTRAN and native R-code. The one-stick-one-ball model has been computed using FSL.

We used the MATLAB toolboxes available at https://sites.google.com/site/pierrickcoupe/softwares/denoising-for-medical-imaging/mri-denoising and http://mr.usc.edu/code.html for processing the data using NLM and the joint denoising method in Haldar et al. (2013), Varadarajan and Haldar (2013), respectively.

We used the MATLAB toolbox available at http://cmic.cs.ucl.ac.uk/mig//index.php?n=Tutorial.NODDImatlab for the estimation of the parameters of the NODDI model.

## Results

### Simulations

For both one-dimensional test functions *θ*_1_(.) and *θ*_2_(.), we show two plots with increasing location bandwidths *h*_1_ = 485,3610 and *h*_2_ = 81,3610 corresponding to the iteration steps *k*_1_ = 30,39 and *k*_2_ = 22,39, see Fig. 3. This illustrates the progress of the estimation function during iteration. The maximal number of iterations is *k*^∗^ = 39 (Figs. 3(b + d)). In the steps *k*_1_ = 30 an *k*_2_ = 22 the mean squared error is minimal (Figs. 3(a + c)).

We may conclude that msPOAS provides the following (heuristic) properties:

• The algorithm separates homogeneous compartments with sufficiently large discontinuities. This allows (almost) consistent estimation of the unknown expected value, which refers to the unknown parameter function. This is illustrated in Figs. 3(a + b), see index values between 1 and 1600 in the local constant example *θ*_1_(.).
• The separation property fails if the discontinuities are small. In this case, the algorithm leads to a bounded estimation bias since different homogeneous compartments are treated as one, see index values between 1601 and 4000 in the same plots as before.
• For model misspecification, i.e., for piecewise smooth parameter functions the estimator is forced into a step function, see the piecewise smooth example *θ*_2_(.) in Figs. 3(c–d). This can be interpreted as an intrinsic stopping criterion of the algorithm as it ensures a bounded estimation bias. Therefore, the maximal location bandwidth $h^{\left(k^{*}\right)}$ and as a consequence the number of iteration steps *k*^∗^ are restricted by the resulting computational workload, only. The choice of *k*^∗^ should ensure a reasonable computation time and provide suitable results within homogeneous regions.

### Experimental data

The multi-shell data was processed by our implementation of msPOAS within 15 min on a single core of a HP SL390s compute server with an Intel Xeon, Six-Core 3467 MHz. The computations using the POAS approach required more than 1 h. This significant acceleration was due to the simplifications and approximations for the discrepancy and the Kullback–Leibler divergence. For the much larger single-shell dataset msPOAS used 3 h and 18 min, while POAS used 4 h and 37 min computation time on a single core of the same machine. Our implementation is parallelized using OpenMP which significantly speeds up the computation compared with these single core results.

**Fig. 4:**
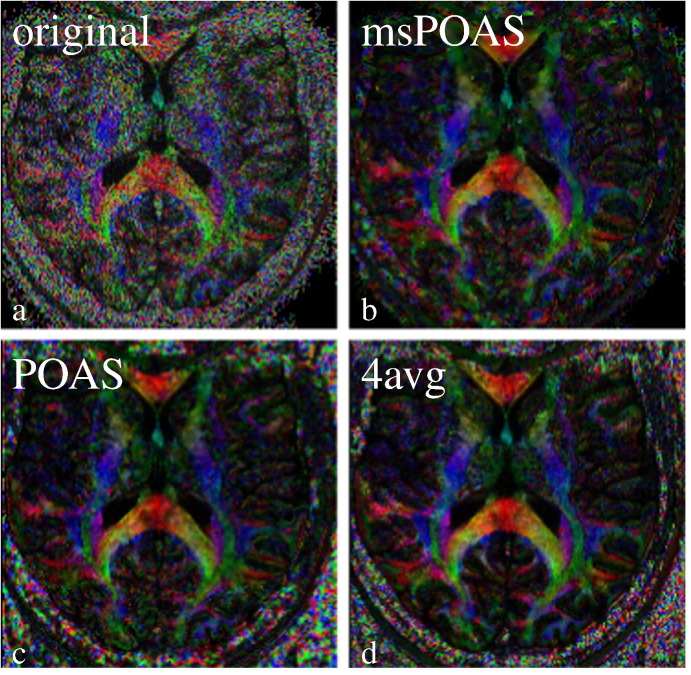
Comparison of color-coded FA maps of the single-shell dataset also used in Becker et al. (2012). a) Original noisy data, b) msPOAS reconstruction, c) POAS reconstruction as in Becker et al. (2012), d) mean image of four repeated measurements from the same session.

We start with the analysis of the first (single-shell) dataset, that was used in Becker et al. (2012) to introduce POAS. In Fig. 4 we show a color-coded FA map for an axial slice of the original data (a). The smoothing effect by msPOAS is obvious in Fig. 4(b), while there is no blurring effect at borders. For comparison we provide the result of the single-shell POAS from Becker et al. (2012) for the same slice which shows quite similar characteristics (c). In Fig. 4(d) we show the color-coded FA obtained from all four (unsmoothed) scans. This image can to a certain degree serve as a high SNR gold standard for the evaluation of the msPOAS (or POAS) result.

**Fig. 5:**
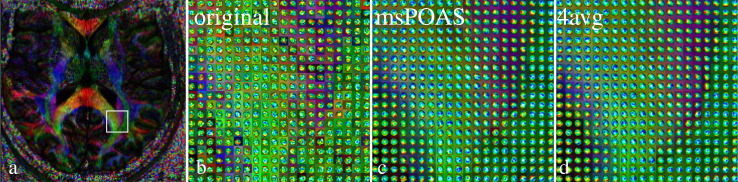
Comparison of the orientation distribution function (ODF) (Descoteaux et al., 2007) in a region-of-interest of an axial slice. a) Position of the region, b) ODF for unsmoothed data, c) ODF for data smoothed by msPOAS, d) ODF from averaged data of all four measurements. In b)–d) the corresponding color-coded FA map from the DTI model is shown as underlay for guidance. The color scheme of the glyphs corresponds to the ODF value.

In Fig. 5 we compare the estimated orientation distribution function following Descoteaux et al. (2007) for the original data, the msPOAS reconstruction and the averaged data from all four measurements.

**Fig. 6:**
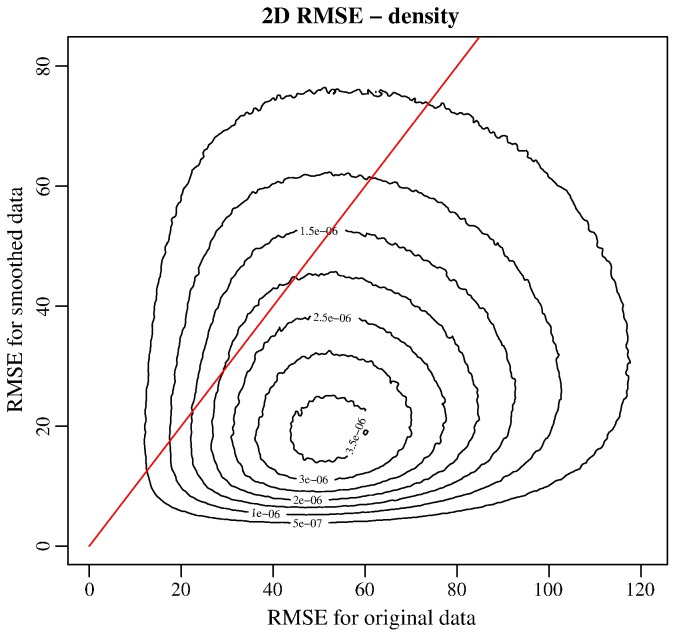
Root mean squared error with respect to the averaged dataset, for original versus reconstructed data using msPOAS.

In Fig. 6 we show the root mean squared error (RMSE) of the data processed by msPOAS with respect to the pseudo-ground truth from the averaged dataset (No. 1) with the corresponding quantity for the original data. This shows, that for most of the voxel the estimate of the data using msPOAS is improved.

**Fig. 7:**
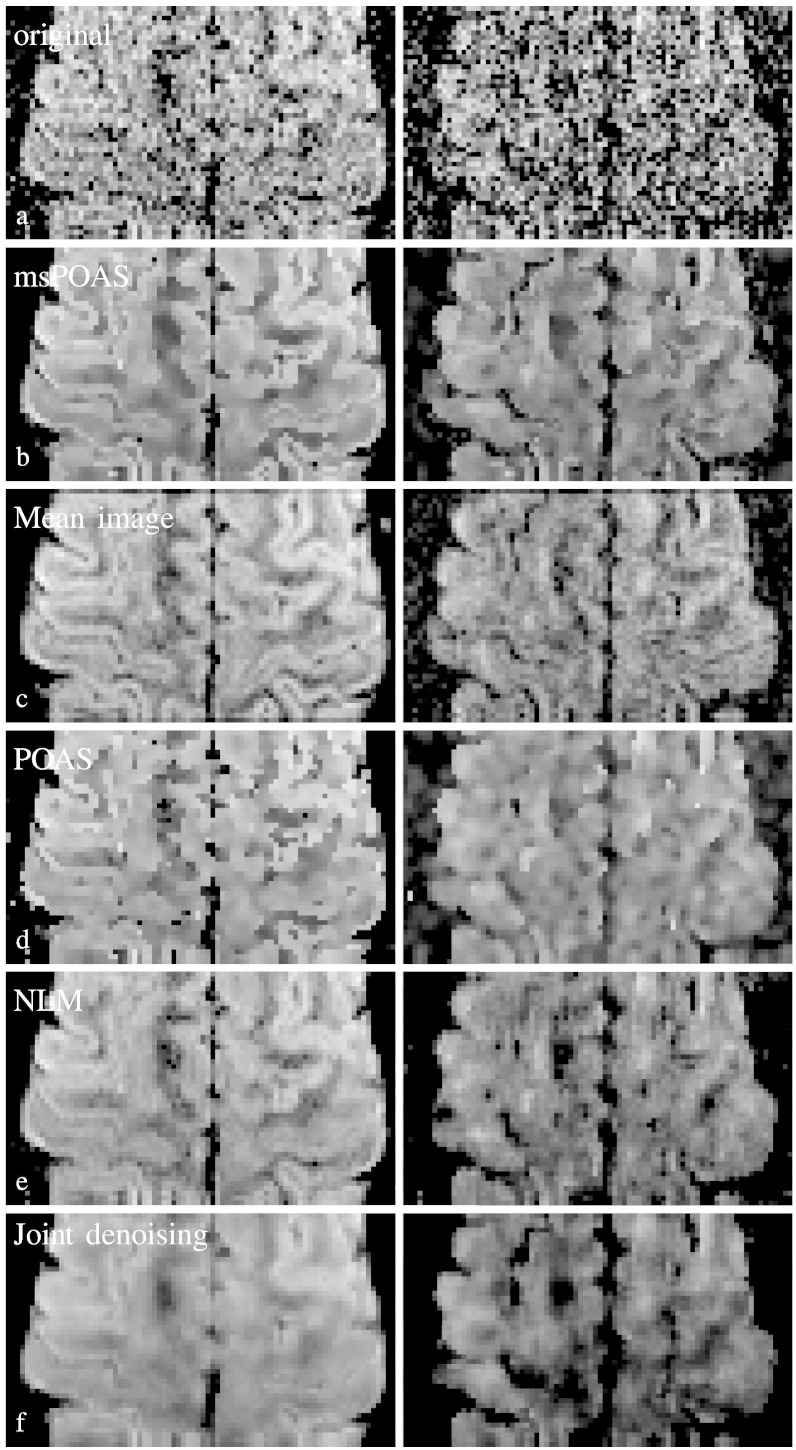
Comparison of the diffusion weighted data for a slice of the double-shell experimental dataset. The left row shows the data at the *b* = 800s/mm^2^-shell, the right row at the *b* = 2000s/mm^2^-shell. The grayscale of the images is set to a maximal image contrast. a) Original noisy data, b) msPOAS reconstruction, c) mean of 10 repeated measurements of the considered diffusion weighted images, d) reconstruction using POAS on each shell separately, e) reconstruction using a non-local means (NLM) method (Wiest-Daesslé et al., 2008), and f) reconstruction using the method described in Haldar et al. (2013), Varadarajan and Haldar (2013).

In Fig. 7 we provide a slice of a diffusion weighted volume for the second double-shell dataset with replicated measurements at both *b*-values *b* = 800 s/mm^2^ (left column) and *b* = 2000s/mm^2^ (right column).

We show the diffusion weighted data before (Fig. 7(a)) and after smoothing using msPOAS (Fig. 7(b)). The adaptive smoothing effect of msPOAS is apparent for both shells. The result of msPOAS is rather robust against misspecification of the effective number of coils *L*′ (not shown here). This is a very helpful property of msPOAS as the estimation of *L*′ from the data is a non-trivial task and the value of *L*′ might not be homogeneous in voxel space. In Fig. 7(c) we show a mean image from 10 repeated measurements of the corresponding diffusion weighted image as ground truth for comparison. In the next row (d) of Fig. 7 we show the result of a POAS analysis. In the last two rows (e) and (f) we report the results of a non-local means filter (Wiest-Daesslé et al., 2008) and the method from Haldar et al. (2013), Varadarajan and Haldar (2013).

**Fig. 8:**
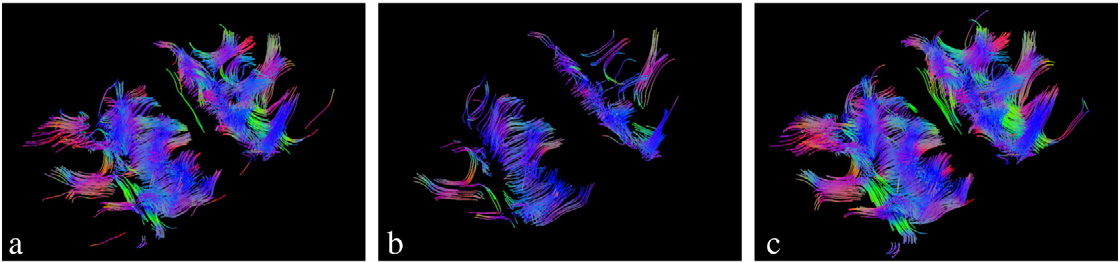
Fiber tracks from a diffusion tensor model of the double-shell data using a FACT algorithm with a minimal fiber length of 25 segments for better visibility. a) Tracks from original data b) TRACKS from POAS reconstruction. c) Tracks after msPOAS reconstruction.

In Fig. 8 we show the fiber track reconstruction for the first double-shell data. Comparison is given for smoothed multi-shell data after the POAS approach, Fig. 8(b), and after msPOAS, Fig. 8(c). We included only fiber tracks with a minimal length of 25 line segments for a better visibility. The figure shows, that after msPOAS the reconstruction of the fibers even with this very simple algorithm was much richer than the one obtained from the POAS approach, see for example the occurrence of the U-Fibers. This confirms the observation from Fig. 7 that msPOAS indeed leads to improved results compared to the POAS approach.

**Fig. 9:**
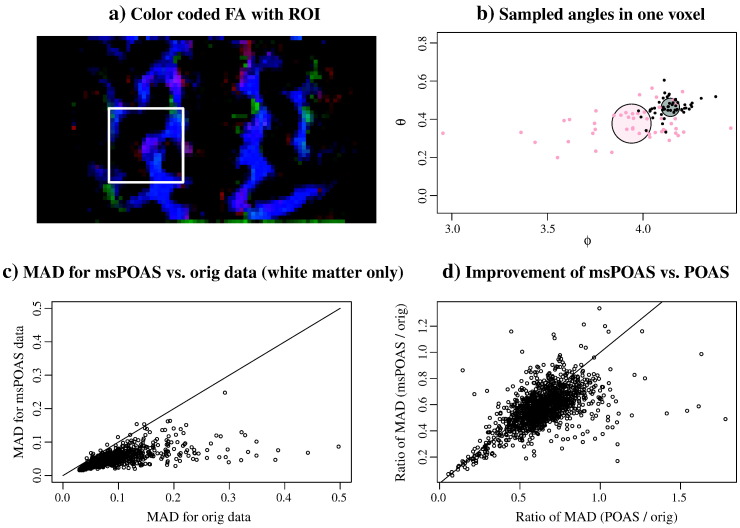
Mean angular deviation (MAD) in a 1-stick-1-ball model from the estimated mean direction of the stick: a) color-coded FA from diffusion tensor model with the region-of interest that has been analyzed, b) sampled “stick directions” expressed in spherical angles *ϕ* and *θ* for the original data (light red) and the msPOAS reconstruction (black) for one white matter voxel, c) comparison of the MAD for the msPOAS reconstruction and for the original data using voxel with a minimal fractional anisotropy of 0.3, and d) comparison of the ratio of the MAD for the msPOAS and the original data with its counterpart from the POAS reconstruction on each shell using voxel with a minimal fractional anisotropy of 0.3.

Finally, we demonstrated quantitatively that directional information estimated from the data was much less variable allowing, e.g., more precise fiber tracking in line with Fig. 8. In Fig. 9 we analyzed the variability of the estimated directions in a one-stick-one-ball model for a small region. A central slice of the region consisting of 10 slices is shown in Fig. 9(a). The white square illustrates the location of the 20 × 20 voxel region-of-interest. In the one-stick-one-ball model a sample of 50 directions for the stick was estimated. In Fig. 9(b) we illustrate for one voxel these directions by plotting them using their representation by spherical angles *ϕ* and *θ*. In light red we show the directions for the original non-smoothed data, in black we show the corresponding points after smoothing with msPOAS. In the following we want to quantify the observation in Fig. 9(b) that these points are much more concentrated for all voxels in the region. We therefore compared the mean angular deviation (MAD) for the sample directions from its mean direction in the original data and after msPOAS reconstruction. For the voxel considered in Fig. 9(b) the circles illustrate the location of the mean direction and this MAD which corresponds to the radius. The mean directions for the original data and the data after msPOAS of course differed. In total, after msPOAS the MAD was much smaller implying that the estimate was much less variable. In Fig. 9(c) we show the improvement for the variability of the directional estimates after msPOAS for all voxel with *FA* > 0.3. The MAD was less after msPOAS for most of the voxel. Finally, we demonstrate, that a slight improvement of msPOAS compared to the POAS approach was found: In Fig. 9(d) we plot the improvement of the MAD compared to the non-smoothed data (ratio) for the POAS method versus the msPOAS approach. Most points lie below the identity line.

**Fig. 10:**
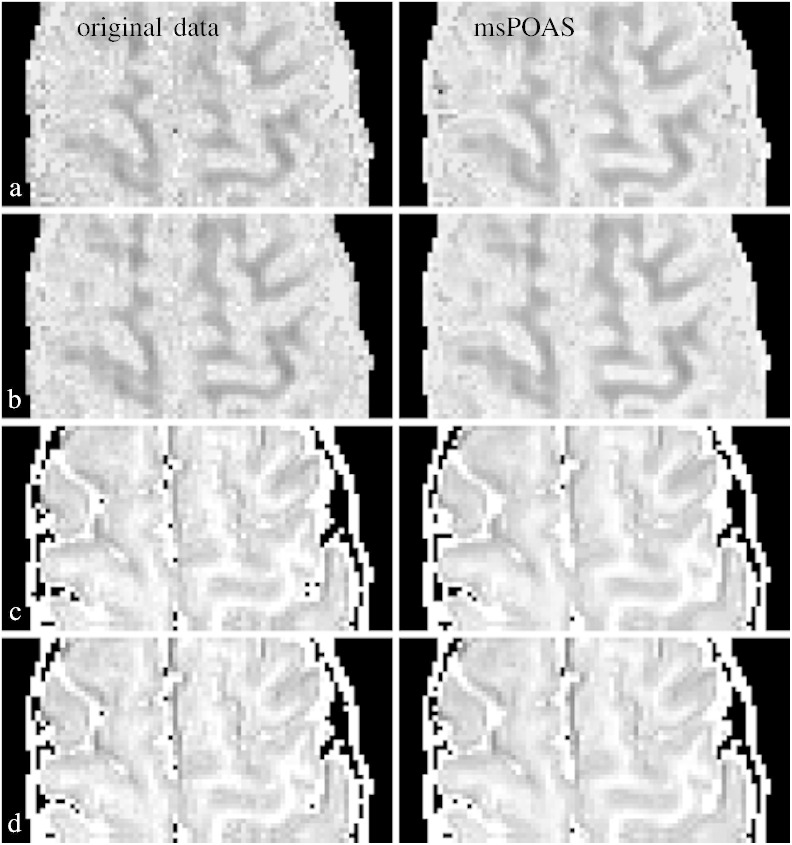
Results from the NODDI model. Left column shows to original data, right column data smoothed by msPOAS. a) Orientation dispersion index (ODI) for the dataset with three shells but using *b* = 800 s/mm^2^ and *b* = 3000 s/mm^2^ only. b) ODI for full dataset. c) Intra-cellular volume fraction (ICVF) for the dataset with three shells but using *b* = 800 s/mm^2^ and *b* = 3000 s/mm^2^ only. d) ICVF for full dataset.

**Fig. 11:**
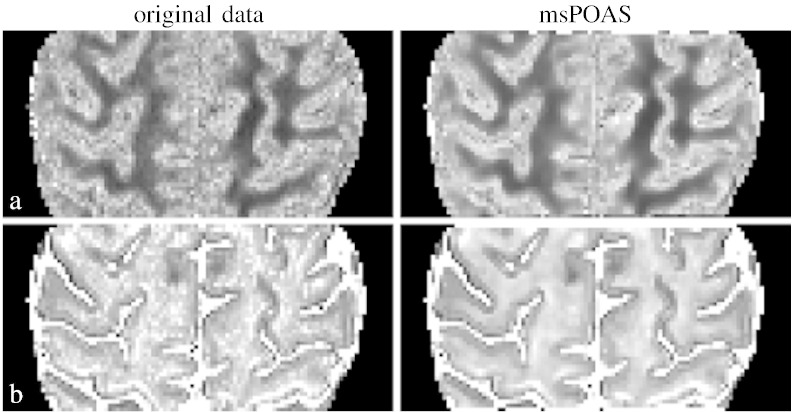
Results from the NODDI model for the double-shell dataset. Left: original data. Right: data smoothed by msPOAS. a) Orientation dispersion index, b) Intra-cellular volume fraction.

In Fig. 10, Fig. 11 we show some results of the evaluation of the NODDI model (Zhang et al., 2012), in particular the orientation dispersion index (ODI) and the intra-cellular volume fraction (ICVF) for two different datasets. In Fig. 10 the dataset with 3 shells (No. 4) was used: Figs. 10(b) and (d) show the considered model indices in a selected central slice for the whole dataset. In contrast the results in Figs. 10(a) and (c) were based on a reduced data set, that contained only the two shells at *b* = 800 s/mm^2^ and *b* = 3000 s/mm^2^. This increased the variability of the model parameter estimates. In Fig. 11 for show the results for the first double-shell dataset at a higher spatial resolution (1.2 mm^3^).

## Discussion

We developed a novel approach for noise reduction in multi-shell diffusion weighted data (msPOAS). It exploits the geometry of the measurement space formed by (voxel) positions and (diffusion sensitizing) orientations as well as the relations of the measurements on the different shells. MsPOAS is applied directly to the diffusion weighted images. It is generally preferable to improve SNR directly on the data, as diffusion model parameters generally suffer from a bias in the observed intensity values complicating smoothing. See e.g. Basser and Pajevic (2000) for a discussion in the context of the diffusion tensor model.

One problem associated with multi-shell dMRI is that the SNR in higher shells is very low. As a result, multi-shell data can only be acquired with low spatial resolution. MsPOAS increases the SNR in multi-shell data and thus enables high resolution multi-shell dMRI.

The method considerably enhances the SNR while it avoids blurring of the tissue micro-structure observed in dMRI by restricting smoothing to (almost) homogeneous compartments. A series of examples with simulated data and real dMRI data demonstrate the effectiveness of msPOAS. The advantages of this approach become obvious when comparing smoothing results of msPOAS with our former procedure POAS for single-shell data, applied to each *q*-shell separately, or other smoothing methods (Fig. 7).

### Comparison of msPOAS and POAS on single-shell data

We demonstrated that msPOAS achieved similar or better results even on single-shell data than POAS, which was explicitly designed for this kind of data. There are of course small differences in detail. MsPOAS for a single shell is not identical to POAS, as information from diffusion weighted and *S*_0_ images is combined to improve adaptivity, see the section on Description of the data. Further differences between our implementations of POAS and msPOAS will be discussed in the section on Relations between msPOAS and POAS.

### Comparison of msPOAS and POAS on multi-shell data

We presented multi-shell dataset with high spatial and angular resolution. We showed that msPOAS indeed reduces the noise in the data without blurring, but also that msPOAS outperforms the POAS approach for each shell and other smoothing methods, see Fig. 7. The reason is that the diffusion weighted signal and contrast at the shells with higher *b*-value are further attenuated. This provides a lower contrast-to-noise ratio (CNR) which complicates adaptation using (single-shell) POAS on higher shells. In contrast, msPOAS uses a vector representation of the data for all shells, and is thus able to achieve the same quality of adaptation on all shells. In other words, it can exploit the higher CNR in low *b*-value shells to inform the adaptive smoothing. We demonstrated this superiority by comparing the smoothed diffusion weighted images, by showing resulting fiber tracks, and by quantitatively analyzing the variability of directional estimates in the sticks and ball model. This demonstrates that even the simple diffusion tensor model benefits from the use of the vector structure of the multi-shell data. While msPOAS further exploits this structure for adaptive smoothing, a separate consideration of the shells may even negatively affect the tensor estimates, since it can introduce inconsistencies between data on different shells.

We also compared the results of msPOAS to a non-local means method (Wiest-Daesslé et al., 2008), a joint denoising method for all diffusion weighted volumes (Haldar et al., 2013, Varadarajan and Haldar, 2013), and a high SNR reference standard from repeated measurements for some selected diffusion directions. Note, that the performance of the method Haldar et al. (2013), Varadarajan and Haldar (2013) may have been somewhat reduced since we did not apply it to *k*-space data.

### Relations between msPOAS and POAS

As msPOAS and POAS are both based on the propagation-separation approach the general behavior is similar for example in case of partial volume effects or in their relation to smoothing methods based on anisotropic diffusion, see the discussion of POAS in Becker et al. (2012, Section 5). MsPOAS draws its additional power from the coupling of data for all *b*-values, including *b* = 0 for adaptation.

In order to simplify and accelerate msPOAS, we introduced several modifications compared to the original POAS approach (Becker et al., 2012): a) Due to computational complexity, the Kullback–Leibler divergence was approximated already in POAS. Here, we introduced a new approximation that further accelerates the computations while also reducing the approximation error (not shown here). Additionally, it motivated the replacement of the weighted quadratic mean in the (non-)adaptive estimator of POAS by a weighted arithmetic mean in msPOAS. b) The new metric for the measurement space provides several useful mathematical properties. It is a pseudo-metric and ensures Euclidean invariance in voxel space and rotational invariance in gradient space. In contrast to the original POAS approach the embedding of ${\mathbb{R}}^{3}\times S^{2}$ into the special Euclidean motion group (Becker et al., 2012) is only needed for the mathematical theory, but no longer for the definition of the algorithm improving accessibility of the method.

MsPOAS also works if other choices for the discrepancy, the spherical interpolation, the distribution of the observations, or the approximation of the Kullback–Leibler distance are made, e.g., to meet specific properties of data. Similarly, the mentioned modifications for msPOAS can be readily used also for POAS.

### Applicability of msPOAS

MsPOAS is a method for noise reduction in dMRI data. It is especially useful in case of low SNR. In these situations modeling the original data by any diffusion model leads to a high variability of their estimated parameters and characteristics. This variability is essentially reduced by the proposed adaptive smoothing procedures without notably compromising the structural information in the data. Note that in case of high SNR the algorithm leaves the data mostly unchanged.

The presented procedure benefits from a high number of measured gradient directions, and also from sampling additional shells. If all shells have an identical gradient scheme, then the algorithm could be further accelerated as no spherical interpolation is needed. In contrast, varying gradient schemes benefit from a higher angular resolution, but possibly suffer from a slightly biased statistical penalty due to the interpolation. In any case, increasing the number of gradients reduces a possible bias introduced by spherical smoothing.

### Estimation of data-dependent parameters

The msPOAS algorithm uses *σ* and *L*′ as data-dependent input parameters. They should be estimated separately by any method that is available and suitable for the data. In this article we assumed homogeneous noise *σ* and effective channel count *L*′ over the voxel space. MsPOAS can be easily adapted to a heteroscedastic situation, at the cost of extended memory usage and computation time.

Generally, if *σ* is underestimated, the adaptation will be very restrictive such that msPOAS does not change the data at all. In contrast, if *σ* is overestimated, oversmoothing occurs with blurring of discontinuities. An accurate estimate for *σ* can therefore improve the results of msPOAS. Note that a misspecification of *σ* can, to some degree, be compensated by adjusting the adaptation parameter λ, see section on Choice of parameters for msPOAS.

MsPOAS has proven to be relatively robust with respect to misspecification of *L*′, which is the parameter that is most difficult to get from image data. Ideally *L*′ should be determined from the parameters and properties of the image reconstruction algorithm. If this information is not available we recommend to use a value considerably smaller than the number of receiver coils for parallel imaging, typically *L*′ = 1,…,4.

### Choice of parameters for msPOAS

The adaptation parameter λ is the crucial parameter of the procedure. It can be chosen independently of the data at hand using simulated data by virtue of a propagation condition detailed in Becker et al. (2013, Section 2.5). This condition determines for a worst case scenario a lower bound of λ with respect to some propagation level ϵ. For instance, the level ϵ = 5 ⋅ 10^− 5^ means, that on average only 5 of 10^5^ estimates would adapt to noise in a homogeneous setting. For this choice of ϵ simulations yield λ = 20, which depends only weakly on the effective number of RF receiver coils *L*′, just as, in our experience, on the number of shells or measured gradients.

To discuss the role of the adaptation bandwidth λ we consider its two extreme cases, λ → *∞* and λ → 0, see Eq. (6). In the former case, the msPOAS result is a non-adaptive estimate as the adaptation term always equals 1. In the latter case, the data will not be changed by msPOAS as the adaptive weights will be zero for any pair of distinct locations in measurement space. Additionally, we observe that λ and the noise standard deviation *σ* influence the procedure in a similar manner. Theoretically, the choice of λ is independent of *σ* due to the considered worst case scenario. However, in practice, subsequent adjustment of λ can improve results by compensating a misspecification of *σ* or other uncertainties that may influence the procedure. Having the general behavior of λ in mind, the adaptation bandwidth can be adjusted using the above choice by the propagation condition as a starting point or, if the propagation level ϵ of λ is sufficiently small, as an upper bound that covers the worst case.

The kernel functions have only a very minor impact on the results and can, e.g., be chosen as in Eq. (2) for efficient computation. When using our implementation in the package **dti** (Tabelow and Polzehl, 2013) this choice is implemented along with the suggested choices of the sequence of bandwidths ${\left\{h^{\left(k\right)}\right\}}_{k=0}^{k^{*}}$ and the balancing parameter sequence *κ*^(*k*)^, see the sections on Initial parameter choices of the algorithm and The non-adaptive weights and the location bandwidths and Appendix B, Initial parameter choices of the algorithm and The non-adaptive weights and the location bandwidths and Appendix B, Appendix B. Then, it remains to fix the initial balancing parameter *κ*_0_ and the maximal location bandwidth $h^{\left(k^{*}\right)}$.

The choice of *κ*_0_ influences the amount of smoothing on the sphere by determining the spherical resolution of msPOAS. For a given total number of applied gradient directions *N_g_* (summed over all shells) the quantity *N*_*g*_(1 − *cos*(*κ*_0_)) is the mean number of neighboring gradients directions with positive weights in the voxel under consideration. We suggest to select *κ*_0_ such that this number is between 5 and 10, i.e., *κ*_0_ depends on the number of measured diffusion-weighting gradients. This leads to a value of *κ*_0_ = 0.3,…,0.6 for the datasets as considered in this paper. Larger values of *κ*_0_ should be chosen in the case of very low SNR in order to stabilize the estimates in the first iteration steps. However, this may introduce a bias. MsPOAS reduces this bias during iteration through its choice of the sequence *κ*^(*k*)^ as explained in our previous article (Becker et al., 2012, Section 2.5). If the number of gradient directions is too low, say less than 20, a much smaller value of *κ*_0_ might be recommendable to avoid spherical smoothing and hence the bias. This requires a sufficient image contrast-to-noise ratio.

The number of iteration steps *k*^⋆^ relates to the last value in the sequence of increasing location bandwidths ${\left\{h^{\left(k\right)}\right\}}_{k=0}^{k^{*}}$. Its choice should balance the computation time and the desired smoothness within homogeneous regions, see section on Simulations. We recommend a value of *k*^⋆^ = 12. As discussed next, possible consequences of a violated structural assumption can be reduced by diminishing *k*^∗^. All parameter choices are summarized in Table 1.

**Table 1:** Choices of parameters for msPOAS.

	Recommendation	Smaller values	Larger values
*σ*	Estimate from data	MsPOAS leaves data unchanged	Blurring
*L*′	1,…,4, see text	Small influence	Small influence
λ	≤ 20	MsPOAS leaves data unchanged	MsPOAS becomes non-adaptive
*κ*_0_	0.3,…,0.6, see text	Reduces spherical bias	Stabilizes estimation, use for small SNR
*k*^⋆^	12	Less noise reduction	Step function approximation

### Considerations and limitations

We should critically discuss the assumption of msPOAS that dMRI data is characterized by regions with homogeneous diffusion weighted signals separated by discontinuities. This assumption is certainly only an approximation of a more realistic piecewise smooth model. In comparison, the application of the common Gaussian filter to diffusion weighted images actually relies on the even stronger assumption of a globally smooth image intensity value. The obvious violation of this assumption in dMRI data manifests itself as the blurring effect at borders when using the Gaussian filter.

As a consequence of the local homogeneity assumption msPOAS forces the final estimator into a step function if the maximal location bandwidth $h^{\left(k^{*}\right)}$ is sufficiently large, see Fig. 3. This figure also illustrates, that intermediate steps of the iteration of msPOAS show results with less error compared to the true situation. Thus, if the msPOAS result contains steps in actually smooth regions, a smaller choice of *k*^∗^ might improve the results. However, even if *k*^∗^ is chosen large, our analysis indicated, that the final stable msPOAS result is a step function approximation providing a bounded estimation bias.

Finally, we emphasize that msPOAS should not be combined with other smoothing methods. Previous application of, e.g., a Gaussian filter would only hamper the performance of msPOAS due to the induced spatial correlation and the resulting blurring. In fact, msPOAS performs very similar to a non-adaptive filter within homogeneous regions, while it does not blur the observed structure at discontinuities.

### Future research

Noise in dMRI data may also lead to a bias in the estimated diffusion model parameters. The quantity characterizing the diffusion weighted signal *θ*_*m*,*b*_ differs from the expected value $ES_{b}\left(m\right)$ of the signal distribution, see Eq. (C.1). It is, however, the expected value, that is actually measured. The relative difference between $ES_{b}\left(m\right)$ and *θ*_*m*,*b*_ is especially large for small SNR. MsPOAS leads to less variable estimates for $ES_{b}\left(m\right)$ and by Eq. (C.1) to largely improved reconstructions for the true parameter *θ*_*m*,*b*_. This enables a correction for the bias, see e.g. Koay and Basser (2006). In practice, this approach requires a precise determination of the (local) noise variance *σ*^2^ and the effectively applied number of receiver coils *L*′, which is a challenging problem in itself and goes beyond the scope of this article.

Additionally, we recall that msPOAS requires independent data in each point of the measurement space. As registration introduces spatial correlation into the data, applying msPOAS before registration may seem preferable. On the other hand, unregistered data might lead to spurious discontinuities, which msPOAS may identify. In our experience, msPOAS benefits from registered data without being harmed by the small spatial correlation caused by it. Future research may combine registration methods with msPOAS to further improve results.

## Conclusion

Multi-shell dMRI acquisition is mandatory for many beyond tensor diffusion models but suffers from low SNR. We introduced the new noise reduction method msPOAS (multi-shell position-orientation adaptive smoothing) for this type of data. The method does not mask the real structure by blurring tissue micro-structure borders. Combining information from all shells for structural adaptive smoothing, it outperforms POAS approaches which smooth each *q*-shell separately or other conventional smoothing. Due to its computational efficiency it can be readily applied. One of the strengths of msPOAS is that it is applied directly to the dMRI data and does not use any diffusion model. Thus, the method does not introduce a bias towards any of them and can be flexibly combined with the various advanced diffusion models. The software packages primarily used for the msPOAS analyses are freely available: **dti** (Tabelow and Polzehl, 2013) and ACID (http://www.diffusiontools.com).

## Appendix A Spherical interpolation

### A.1 Definition of the interpolation weights

Let *b* ∈ *B* be a fixed *b*-value and consider a gradient direction $\vec{g}\notin G_{b}$, that has not been included in the measurement on the corresponding shell. Then, we search for a triple of measured gradients ${\left\{g_{\left(b,\vec{g}\right)}^{\left(l\right)}\right\}}_{l=1}^{3}\subseteq G_{b}$, such that the gradient direction $\vec{g}$ lies within the resulting spherical triangle

$$\vec{g}\in △\left(g_{\left(b,\vec{g}\right)}^{\left(1\right)},g_{\left(b,\vec{g}\right)}^{\left(2\right)},g_{\left(b,\vec{g}\right)}^{\left(3\right)}\right)$$

and the corresponding total angular distance $\sum_{l=1}^{3}\arccos\left(\langle \vec{g},g_{\left(b,\vec{g}\right)}^{\left(l\right)}\rangle \right)$ is minimal. Here, 〈⋅,⋅〉 denotes the Euclidean scalar product of two vectors. The missing value $S_{b}\left(\vec{v},\vec{g}\right)$ is then generated by linear spherical interpolation

$$S_{b}\left(\vec{v},\vec{g}\right):=\sum_{l=1}^{3}\;c_{\left(b,\vec{g}\right)}^{\left(l\right)}S_{b}\left(\vec{v},g_{\left(b,\vec{g}\right)}^{\left(l\right)}\right)$$

with weights $c_{\left(b,\vec{g}\right)}^{\left(l\right)}$ defined as the spherical Bary-coordinates (Carfora, 2007, 3.1(c)) of $\vec{g}$ within the spherical triangle $△\left(g_{\left(b,\vec{g}\right)}^{\left(1\right)},g_{\left(b,\vec{g}\right)}^{\left(2\right)},g_{\left(b,\vec{g}\right)}^{\left(3\right)}\right)$. For *l* ∈ {1, 2, 3} we set

$$c_{\left(b,\vec{g}\right)}^{\left(l\right)}:=\frac{\mathrm{area}\left(△\left(\vec{g},g_{\left(b,\vec{g}\right)}^{\left(l_{1}\right)},g_{\left(b,\vec{g}\right)}^{\left(l_{2}\right)}\right)\right)}{\mathrm{area}\left(△\left(g_{\left(b,\vec{g}\right)}^{\left(1\right)},g_{\left(b,\vec{g}\right)}^{\left(2\right)},g_{\left(b,\vec{g}\right)}^{\left(3\right)}\right)\right)}\;\mathrm{where}\;\begin{array}{c} l_{1},l_{2}\in \left\{123\right\}\setminus \left\{l\right\},\\ l_{1}\neq l_{2}. \end{array}$$

This interpolation is illustrated in Becker et al. (2013, Figure 2).

### A.2 Interpolation of the signal vector

In our vector-valued function S, Eq. (1), we distinguish between values $S_{b}\left(\vec{v},\vec{g}\right)$ that have been measured $\left(\vec{g}\in G_{b}\right)$, values that have been obtained by interpolation $\left(\vec{g}\in G_{b^{\prime }}\setminus G_{b},b^{\prime }>0\right)$ and mean signals for $\vec{g}\in G_{0}$, such that

(A.1) $$S_{b}\left(\vec{v},\vec{g}\right)=\left\{\begin{array}{ll} S_{b}\left(\vec{v},\vec{g}\right), & \mathrm{if}\;\vec{g}\in G_{b}\\ \sum_{l=1}^{3}c_{\left(b,\vec{g}\right)}^{\left(l\right)}S_{b}\left(\vec{v},g_{\left(b,\vec{g}\right)}^{\left(l\right)}\right), & \mathrm{if}\;\vec{g}\in G_{b^{\prime }}\setminus G_{b},b^{\prime }>0\\ |G_{b}|{}^{-1}\sum_{\vec{g}\in G_{b}}S_{b}\left(\vec{v},\vec{g}\right) & \mathrm{if}\;\vec{g}\in G_{0}. \end{array}\right)$$

Estimates in msPOAS are constructed as weighted averages of the observed values ${\left\{S_{b}\left(\vec{v},\vec{g}\right)\right\}}_{\vec{g}\in G_{b}}$. The interpolated values ${\left\{S_{b}\left(\vec{v},\vec{g}\right)\right\}}_{\vec{g}\in G\setminus G_{b}}$ are solely used to determine the adaptive weights, see section on The algorithm.

### A.3 Consequences for the adaptive estimator

Taking the impact of the spherical interpolation into account, we consider for ${\tilde{N}}_{m,b}^{\left(k-1\right)}$ in Eq. (5) the same classification of cases as for the interpolation formula (A.1). For *b* = 0, we down-weight the influence of the *S*_0_-images to compensate for the variance reduction from averaging all acquired non-diffusion weighted images. Hence, for $m=\left({\vec{v}}_{m},{\vec{g}}_{m}\right)$ we set

(A.2) $${\tilde{N}}_{m,b}^{\left(k\right)}=\left\{\begin{array}{ll} \max_{k^{\prime }\leq k}\left(\sum_{n\in V\times G_{b}}{\tilde{w}}_{\mathrm{mn}}^{\left(k^{\prime }\right)}\right) & \mathrm{if}\;b>0\wedge {\vec{g}}_{m}\in G_{b},\\ \max_{k^{\prime }\leq k}{\left(\sum_{l=1}^{3}c_{b,{\vec{g}}_{m}}^{\left(l\right)}/{\tilde{N}}_{\left({\vec{v}}_{m},g_{b,{\vec{g}}_{m}}^{\left(l\right)}\right),b}^{\left(k^{\prime }\right)}\right)}^{-1} & \mathrm{if}\;b>0,{\vec{g}}_{m}\in G_{b^{\prime }},0<b^{\prime }\neq b,\\ \max_{k^{\prime }\leq k}{\left(\sum_{\vec{g}\in G_{b}}1/{\tilde{N}}_{\left({\vec{v}}_{m},\vec{g}\right),b}^{\left(k^{\prime }\right)}\right)}^{-1}\cdot |G_{b}| & \mathrm{if}\;b>0\wedge {\vec{g}}_{m}\in G_{0},\\ \max_{k^{\prime }\leq k}\left(\sum_{n\in V\times G_{0}}{\tilde{w}}_{\mathrm{mn}}^{\left(k^{\prime }\right)}\right)\cdot {\left|S_{0}\right|}^{-1} & \mathrm{if}\;b=0\wedge {\vec{g}}_{m}\in G_{b} \end{array}\right)$$

where |*G*_*b*_| denotes the number of measured gradients on the shell with *b*-value *b* and |*S*_0_| is the number of acquired *S*_0_-images that form the mean image *S*_0_. Note, that we consider the maximal variance reduction ${\tilde{N}}_{m,b}^{\left(k\right)}:=\max_{k^{\prime }\leq k}\left(\cdot \right)$ until step *k* in order to ensure that the sequence ${\left\{{\tilde{N}}_{m,b}^{\left(k\right)}\right\}}_{k=0}^{k^{*}}$ is non-decreasing. This preserves an achieved adaptation quality during iteration.

## Appendix B Choice of the sequence of bandwidths

The bandwidths *h* are measured in units of voxel counts with respect to the smallest of the three voxel edge lengths. We choose *h*^(0)^ = 1, i.e., one unit of the shortest edge of the voxel. In our implementation of msPOAS, we chose the subsequent bandwidths ${\left\{h^{\left(k\right)}\right\}}_{k=1}^{k^{*}}$ such that the variance of the non-adaptive estimate is, in the interior of the design space *V* × *G*, reduced by 25% with each iteration step. In our experience this provides a good balance between the quality of adaptation and computation time. We employ the regular grid structure of the voxel space *V* to pre-compute the non-adaptive weights ${\bar{w}}_{\mathrm{mn}}^{\left(k\right)}$ for some representative voxel ${\vec{v}}_{m}\in V$, all *b* ∈ *B*_0_ and every ${\vec{g}}_{m}\in G_{b}$ using a sequence of location bandwidths ${\left\{h^{\left(k\right)}\left(b,{\vec{g}}_{m}\right)\right\}}_{k=1}^{k^{*}}$ that solves

$$\sum_{n\in V\times G_{b}}\frac{{\left({\bar{w}}_{\mathrm{mn}}^{\left(k\right)}\right)}^{2}}{{\left({\bar{N}}_{m,b}^{\left(k\right)}\right)}^{2}}=1.25^{-1}\sum_{n\in V\times G_{b}}\frac{{\left({\bar{w}}_{\mathrm{mn}}^{\left(k-1\right)}\right)}^{2}}{{\left({\bar{N}}_{m,b}^{\left(k-1\right)}\right)}^{2}},$$

where $m=\left({\vec{v}}_{m},g_{m}\right)\in V\times G_{b}$. As the number of applied gradients may vary for the distinct shells, this leads to a choice of ${\left\{h^{\left(k\right)}\right\}}_{k=0}^{k^{*}}$ which depends on the considered *b*-value *b* ∈ *B*_0_ via the respective gradient scheme $G_{b}∍{\vec{g}}_{m}$. Additionally, we get a dependence on the gradient ${\vec{g}}_{m}$ itself by compensating possible inhomogeneities of the gradient scheme. As a consequence our choice for *κ*^(*k*)^ now also depends on *b* ∈ *B*_0_ and ${\vec{g}}_{m}\in G_{b}$. The resulting weights depend on the relative positions of ${\vec{v}}_{m}$ and ${\vec{v}}_{n}$ on the grid and can therefore be used for all voxel ${\vec{v}}_{m}\in V$.

## Appendix C Approximation of the Kullback–Leibler divergence

The Kullback–Leibler divergence of two non-central *χ* -distributions cannot be calculated explicitly. In order to achieve a much faster performance we replace the approximation utilized in Becker et al. (2012). We approximate $\chi_{2L^{\prime }}\left(\theta \right)$ by a Gaussian distribution with matching first and second moments, i.e. mean

(C.1) $$\mu \left(\theta ,2L^{\prime }\right)=\sqrt{\frac{\pi }{2}}L_{1/2}^{\left(L^{\prime }-1\right)}\left(-\frac{\theta^{2}}{2}\right)$$

and variance

$$v\left(\theta ,2L^{\prime }\right)=2L^{\prime }+\theta^{2}-\mu^{2}\left(\theta ,2L^{\prime }\right)$$

depending on the non-centrality parameter *θ*.

Then, we replace the Kullback–Leibler divergence of two non-central *χ*-distributions $\chi_{2L^{\prime },\theta_{1}}$ and $\chi_{2L^{\prime },\theta_{2}}$ by

$$\tilde{KL}\left(\chi_{2L^{\prime },\theta_{1}},\chi_{2L^{\prime },\theta_{2}}\right)=\frac{2{\left[\mu \left(\theta_{1},2L^{\prime }\right)-\mu \left(\theta_{2},2L^{\prime }\right)\right]}^{2}}{\left[\left(v\left(\theta_{1},2L^{\prime }\right)+v\left(\theta_{2},2L^{\prime }\right)\right)\right]},$$

which can be interpreted as a symmetrized Kullback–Leibler divergence of the approximating Gaussian distributions.

## Appendix D Supplementary data

Supplementary video showing the development of the weights over iterations for non-adaptive and adaptive smoothing.
